# ALDH1A1 Maintains Ovarian Cancer Stem Cell-Like Properties by Altered Regulation of Cell Cycle Checkpoint and DNA Repair Network Signaling

**DOI:** 10.1371/journal.pone.0107142

**Published:** 2014-09-12

**Authors:** Erhong Meng, Aparna Mitra, Kaushlendra Tripathi, Michael A. Finan, Jennifer Scalici, Steve McClellan, Luciana Madeira da Silva, Eddie Reed, Lalita A. Shevde, Komaraiah Palle, Rodney P. Rocconi

**Affiliations:** 1 University of South Alabama Mitchell Cancer Institute, Mobile, Alabama, United States of America; 2 National Institutes of Health, National Institute on Minority Health and Health Disparities, Bethesda, Maryland, United States of America; 3 University of Alabama at Birmingham, Birmingham, Alabama, United States of America; University College London, United Kingdom

## Abstract

**Objective:**

Aldehyde dehydrogenase (ALDH) expressing cells have been characterized as possessing stem cell-like properties. We evaluated ALDH+ ovarian cancer stem cell-like properties and their role in platinum resistance.

**Methods:**

Isogenic ovarian cancer cell lines for platinum sensitivity (A2780) and platinum resistant (A2780/CP70) as well as ascites from ovarian cancer patients were analyzed for ALDH+ by flow cytometry to determine its association to platinum resistance, recurrence and survival. A stable shRNA knockdown model for ALDH1A1 was utilized to determine its effect on cancer stem cell-like properties, cell cycle checkpoints, and DNA repair mediators.

**Results:**

ALDH status directly correlated to platinum resistance in primary ovarian cancer samples obtained from ascites. Patients with ALDH^HIGH^ displayed significantly lower progression free survival than the patients with ALDH^LOW^ cells (9 vs. 3 months, respectively p<0.01). ALDH1A1-knockdown significantly attenuated clonogenic potential, PARP-1 protein levels, and reversed inherent platinum resistance. ALDH1A1-knockdown resulted in dramatic decrease of KLF4 and p21 protein levels thereby leading to S and G2 phase accumulation of cells. Increases in S and G2 cells demonstrated increased expression of replication stress associated Fanconi Anemia DNA repair proteins (FANCD2, FANCJ) and replication checkpoint (pS317 Chk1) were affected. ALDH1A1-knockdown induced DNA damage, evidenced by robust induction of γ-H2AX and BAX mediated apoptosis, with significant increases in BRCA1 expression, suggesting ALDH1A1-dependent regulation of cell cycle checkpoints and DNA repair networks in ovarian cancer stem-like cells.

**Conclusion:**

This data suggests that ovarian cancer cells expressing ALDH1A1 may maintain platinum resistance by altered regulation of cell cycle checkpoint and DNA repair network signaling.

## Introduction

Ovarian cancer is the most lethal of all gynecologic malignancies, affecting over 22,000 lives of women annually in the United States alone. Although the majority of ovarian cancer patients achieve a complete initial clinical response to cytoreductive surgery followed by combination chemotherapy, most will experience a recurrence and unfortunately succumb to progressive disease [1]. Vital to the prognosis of ovarian cancer patients is the disease’s varying sensitivity to platinum agents. Although a continuum, patients are stratified by their disease’s original response to platinum chemotherapy as either “platinum-sensitive” or “platinum-resistant” defined by the length of the relapse-free interval. This spectrum is highly predictive of clinical endpoints of when a cancer recurs, the success of surgery and/or chemotherapy at recurrence, and a patient’s overall survival.

Considering the heterogeneity of cancer, not all cells within a malignancy would be expected to be resistant to chemotherapy. The cancer stem cells (CSCs) theory proposes that these resistant cells encompass only a minority of cells within a cancer, yet are solely responsible for long-term recurrence [2]. Thereby, irrespective of the initial response rates, if chemotherapy fails to eradicate these resistant CSCs, then cancer will regenerate and a recurrence or progression of disease will occur. The identification of these resistant cells and determining their innate molecular pathways are paramount in finding more effective targeted therapies [3]. Therefore, one strategy to improve the success of ovarian cancer therapy is to enhance CSCs sensitivity to platinum agents. Overcoming platinum resistance would be vital in the treatment of ovarian cancer with the potential benefits of enhanced response rates, longer survival, and more cures.

Recently, aldehyde dehydrogenase (ALDH) activity has been shown to be a very attractive CSCs marker in many cancers such as lung [4], breast [5], prostate [6], thyroid [7], head and neck cancer [8], and ovarian cancer [9]–[12]. ALDH family comprises cytosolic isoenzymes responsible for oxidizing intracellular aldehydes, thus contributing to the oxidation of retinol to retinoic acid in early stem cell differentiation [4]. The human ALDH superfamily currently consists of 19 known putatively functional genes in 11 families and 4 subfamilies with distinct chromosomal locations. Of the vast ALDH families and subfamilies, ALDH1A1 has been a valid marker among several malignant tissues. It holds the attractive distinction of not only being a potential marker of stemness but potentially playing a role in the biology of tumor-initiating cells as well [13]. Additionally, the ALDH1A1 subpopulation had demonstrated to be associated with chemoresistance in ovarian cancer patients [9], [14].

Recent studies in breast cancer models demonstrated an interesting relationship between BRCA1 and stem cell differentiation [15], [16]. BRCA1 also has been shown to play an important role in breast tissue differentiation by regulating Notch signaling and tumor response to anti-endocrine therapy[14]. Particularly, an inverse relationship between ALDH1A1 expression and BRCA1 is noteworthy in the context of studying cancer stem-like cells and chemoresistance. BRCA1 plays important roles in protecting genome from aberrant DNA lesions, and mutations or deletion in this gene lead to genome instability and increased incidence of breast, ovarian and other cancers [17]. In response to DNA damage it rapidly localizes to the sites of double strand breaks (DSB) and mediates several signaling responses including cell cycle checkpoints and choice of the DNA repair pathway to fix these lesions [17]–[19]. However, BRCA1 status and ALDH+ ovarian cancer stem-like cells maintenance and their resistance to chemotherapy has not been studied.

We demonstrate that ALDH+ phenotype possesses CSC characteristics of enhanced invasion, colony formation, and stem cell markers. The specific ALDH1A1 isotype appears to be responsible for ALDH-mediated platinum resistance both clinically as well as *in in-vitro* models. Our data supports an ALDH1A1-mediated platinum resistance mechanism in ovarian cancer via an altered regulation of cell cycle checkpoint and DNA repair network signaling.

## Materials and Methods

### Cell Lines and Cultures

A2780 and an isogenic cisplatin resistant A2780/CP70 cell line was generated as described earlier [20].

### ALDEFLUOR Assay and Fluorescence-activated Cell Sorting

To isolate the cell population with a high ALDH enzymatic activity, ALDEFLUOR assay kit (STEMCELL Technologies Inc.) was used according to the manufacturer’s instructions. After trypsinization, cells were suspended in ALDEFLUOR assay buffer containing ALDH enzyme substrate BODIPY-aminoacetaldehyde (BAAA), and incubated at 37°C for about 40 minutes. Cells were stained using the identical conditions with the specific ALDH inhibitor, diethylaminobenzaldehyde (DEAB), to serve as a negative control. Flow cytometry sorting was conducted using a BD Bioscience Aria II SORP cell sorter.

### Carcinogenic Properties

After sorting for ALDH phenotypes (ALDH+ vs. ALDH−), Matrigel invasion and soft agar colony formation assays were performed as previously described [21]. To evaluate the effect of chemotherapy on colony formation, cells were treated with fresh media with 20 µM carboplatin added every 3–4 days. Visible colonies (>50 cells) were counted on five randomly selected 40X microscopic fields in each well.

### CellTiter-Glo Luminescent Cell Viability Assay

Sorted A2780/CP70 cells were plated at density of 4000 cells per well in 96-well black plate with clear bottom (Corning, NY, USA). The following day, the cells were treated with various concentrations of carboplatin up to 72 hours. After 24, 48 and 72 hours, 100 µl of CellTiter-Glo reagent (Promega) per well were added, incubated for 10 minutes at room temperature and luminescence was recorded on a Synergy H4 Hybrid Reader (BioTek).

### Real-time Quantitative RT-PCR

Real-time Quantitative RT-PCR was performed as described previously [21]. Primers and probes for the TaqMan system were selected from the Applied Biosystems website [BMI1 assay ID: Hs00180411_m1, c-myc assay ID: Hs00905030_m1, Kruppel-like factor 4 (KLF4) assay ID: Hs00358836_m1, oct3/4 assay ID: Hs01009568_m1, S100 calcium binding protein A1 (S100A1) assay ID: Hs00984741_m1, ALDH1A1 assay ID: Hs00946916_m1, CDKN1A (p21) assay ID: Hs00355782_m1, internal control glyceraldehyde-3-phosphate dehydrogenase assay ID: Hs99999905_m1]. The relative expression mRNA levels of BMI1, c-myc, Oct3/4, KLF4, S100A1, ALDH1A1, p21, were calculated using the ΔΔCt method and normalization to GAPDH.

### Lentiviral shRNA Vector Transfection

Six different pGIPZ Lentiviral shRNA glycerol stocks against ALDH1A1 and one negative control shRNA (Thermo Scientific) were transfected according to the manufacturer’s instructions. A2780/CP70 cells were plated at a density of 2×10^5^ cells per well of a 6-well plate for 18–24 hours. For each well, 2 µg shRNA plasmid DNA (pGIPZ) were transfected into A2780/CP70 using Arrest-In reagent (Thermo Scientific, USA). Puromycin (0.8 µg/ml) was used to select the transfected cells. After optimization, ALDH1A1-knockdown efficiencies of individual shRNAs were evaluated using real-time quantitative PCR and Western Blot. Vector 398453 demonstrated the most efficient transfection with over 95% knockdown efficacy of ALDH1A1 and was used for all shRNA knockdown experiments.

### Western Blot Analysis

Cultured cells were collected in NP-40 lysis buffer with 10 µl/ml protease inhibitor cocktail (Sigma) and subjected to immunoblotting analysis by standard techniques using antibodies against ALDH1A1, FANCJ, KLF4 (Sigma-Aldrich), FANCD2, PARP-1, XRCC1, β-actin, GAPDH (Santa Cruz Biotechnology, Inc.), and p21, phospho–Chk1 (Ser317), BRCA1 antibodies (Cell Signaling Technology).

### RT^2^ Profiler PCR Array

The human Cancer Drug Resistance & Cell Cycle RT^2^ Profiler PCR Array (Qiagen, USA) was used to profile the expression of 84 genes involved in cancer drug resistance and cell cycle with five housekeeping genes per manufacturer’s instructions. Controls for genomic DNA contamination and for the efficiency of the RT-PCR and PCR reactions were also assayed. The PCR arrays were run using a Bio-Rad iQ5 real-time detection system (Bio-Rad).

### Cell Cycle Analysis by Flow Cytometry

A2780/CP70 cells were transfected with control or shRNAs targeting to ALDH1A1 using Lipofectamine 2000 (Life Technologies) and cells were collected and stained for cell cycle analysis according to the manufacturer’s instructions (BD Biosciences). Cells at 10^6^ were re-suspended with 300 µl PBS, and 700 µl ice cold methanol to fix the cells for overnight at −20°C. Cells were washed twice with PBS, and then stained with 500 µl propidium iodide (BD Bioscience) at room temperature for 30 minutes in the dark. Cell cycle profiles were analyzed by flow cytometry.

### Apoptosis Assay

A2780/CP70 cells transfected with negative control or shRNA-ALDH1A1 were induced for apoptosis by treating with 1.0 µM staurosporine for 6 hours [22], [23]. APC-Annexin V and 7-AAD staining was performed according to the manufacturer’s instructions. Each group contains three isotype controls: unstained cells; cells stained with APC Annexin V alone; cells stained with 7-AAD alone. Samples were analyzed by flow cytometry.

### PARP Activity Assay

PARP activity was measured using HT PARP in vivo Pharmacodynamic Assay II kit (Trevigen). After treatment with 100 µM carboplatin for 45 minutes, A2780/CP70 cells transfected with negative control or shRNA-ALDH1A1 were washed twice with 5 ml of warm (37°C) PBS. Cells were scraped into 300 µl of cold cell lysis buffer and incubated on ice for 15 minutes. SDS was added to samples to a final SDS concentration of 1% and incubated at 100°C for 5 minutes. When samples cooled to room temperature, 0.01 volume of 100X Magnesium cation and 2 µl of DNase I (2 Units/µl) were added to the samples and incubated for additional 90 minutes at 37°C. After a brief centrifugation, supernatants were collected and polyADP-ribose (PAR) levels in the cell extracts were quantified using ELISA method.

### Clinical Correlation

All the participants provided their written informed consent to participate in this study and the institutional review board at the University of South Alabama Health System approved this study, as well as the consent procedure. Ascites from 15 consecutive patients undergoing surgery for advanced stage IIIC/IV papillary serous ovarian cancer as well as 2 patients with benign ascites were collected and was immediately processed to perform ALDEFLUOR assay after washing them with PBS and removing the erythrocytes using ACK lysing buffer (Lonza, Walkersville, MD, USA). Clinicopathologic data was collected for the respective patients and correlated to percentage of cells exhibited ALDH+ phenotype in their ascites.

### Statistical Analysis

Comparisons between two groups were carried out using Student’s t-test and ANOVA where appropriate. Statistical significance was determined at *p*<0.05. Kaplan-Meier curve was performed for survival with log-rank for statistical comparison.

## Results

### ALDH status correlates with ovarian cancer resistance to platinum agents

**Figure 1:**
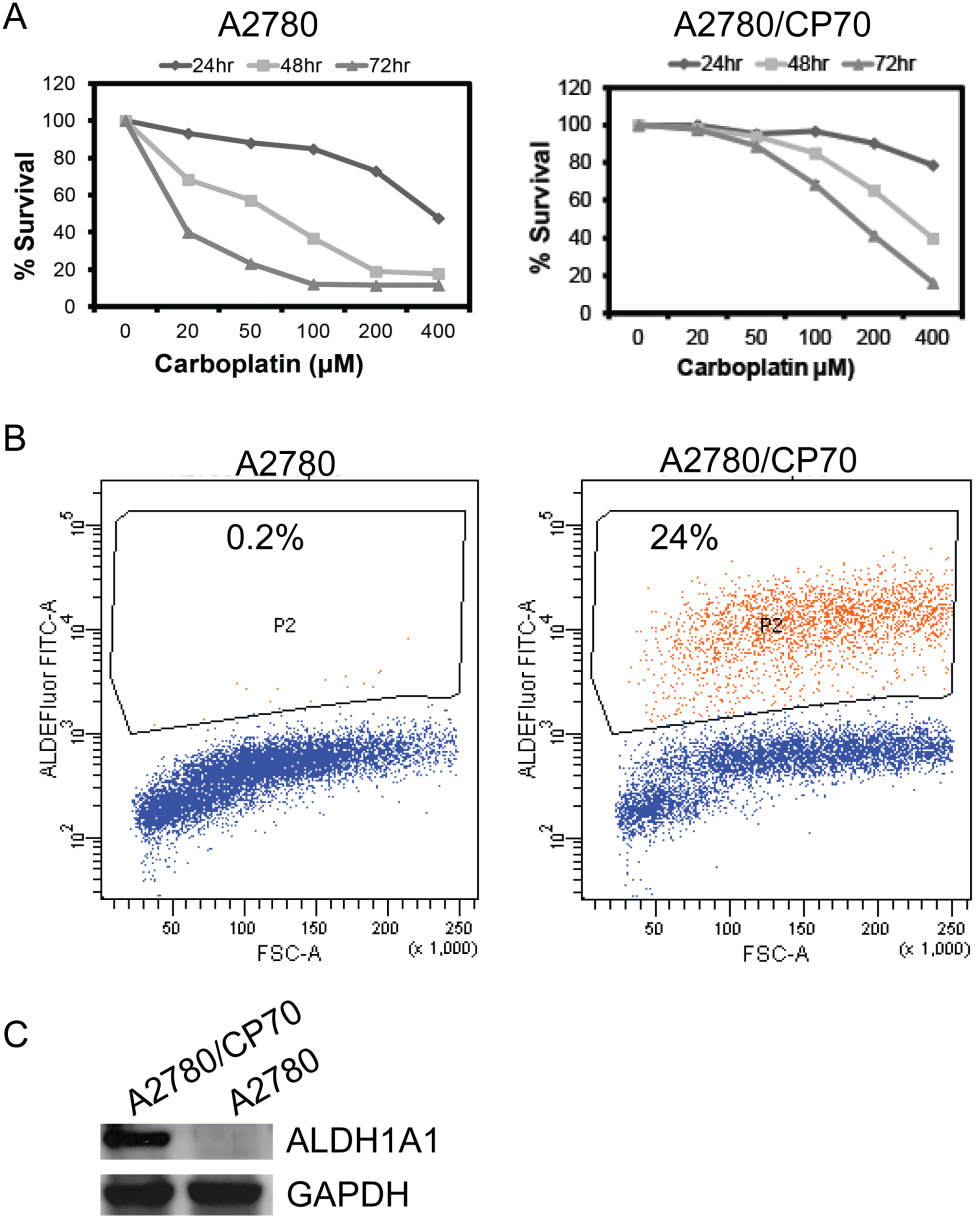
ALDH1A1 status correlates with platinum resistance in ovarian cancer cells. Isogenic cell lines (A2780- platinum sensitive & A2780/CP70- platinum resistant) were evaluated for platinum response using drug sensitivity assay as described in M&M section (A). To assess the percent of ALDH+ cells, in A2780 and A2780/CP70 cells ALDEFLUOR assay was performed by using BODIPY-aminoacetaldehyde (BAAA) as substrate for ALDH enzyme, after 40 minutes incubation at 37°C flow cytometry analysis was performed (B) and western blot data shows representing levels of ALDH1A1 isozyme in these cells (C).

Isogenic cell lines of platinum sensitive (A2780) and resistant (A2780/CP70) ovarian cancer cells were evaluated for their survival rates in the presence and absence of different doses of carboplatin. Consistent with the previously reported results [24], [25], A2780/CP70 cells required up to a 10-fold higher dose of carboplatin to achieve the IC_50_ concentration compared to its platinum sensitive counterpart, A2780 (Figure 1A). Recently, in several cancer models, ALDH status has been implicated in tumor resistance to chemotherapy by maintaining cancer stem-like cells’ characteristics such as aggressive growth, increased survival and re-differentiation potential [9]. In order to test the correlation between ALDH status and platinum resistance in ovarian cancer cells, ALDEFLOUR assays were performed on these isogenic cells. Interestingly platinum resistant ovarian cancer cell line A2780/CP70 exhibited at least 110-fold higher percentage of ALDH+ cells (with a range of 22–40%) compared to the platinum sensitive A2780 cells, which had only 0.2% ALDH+ cells (Figure 1B). Likewise, western blot analysis of ALDH1A1 protein levels in these cells revealed similar results, suggesting association of ALDH status with platinum resistance (Figure 1C).

**Figure 2:**
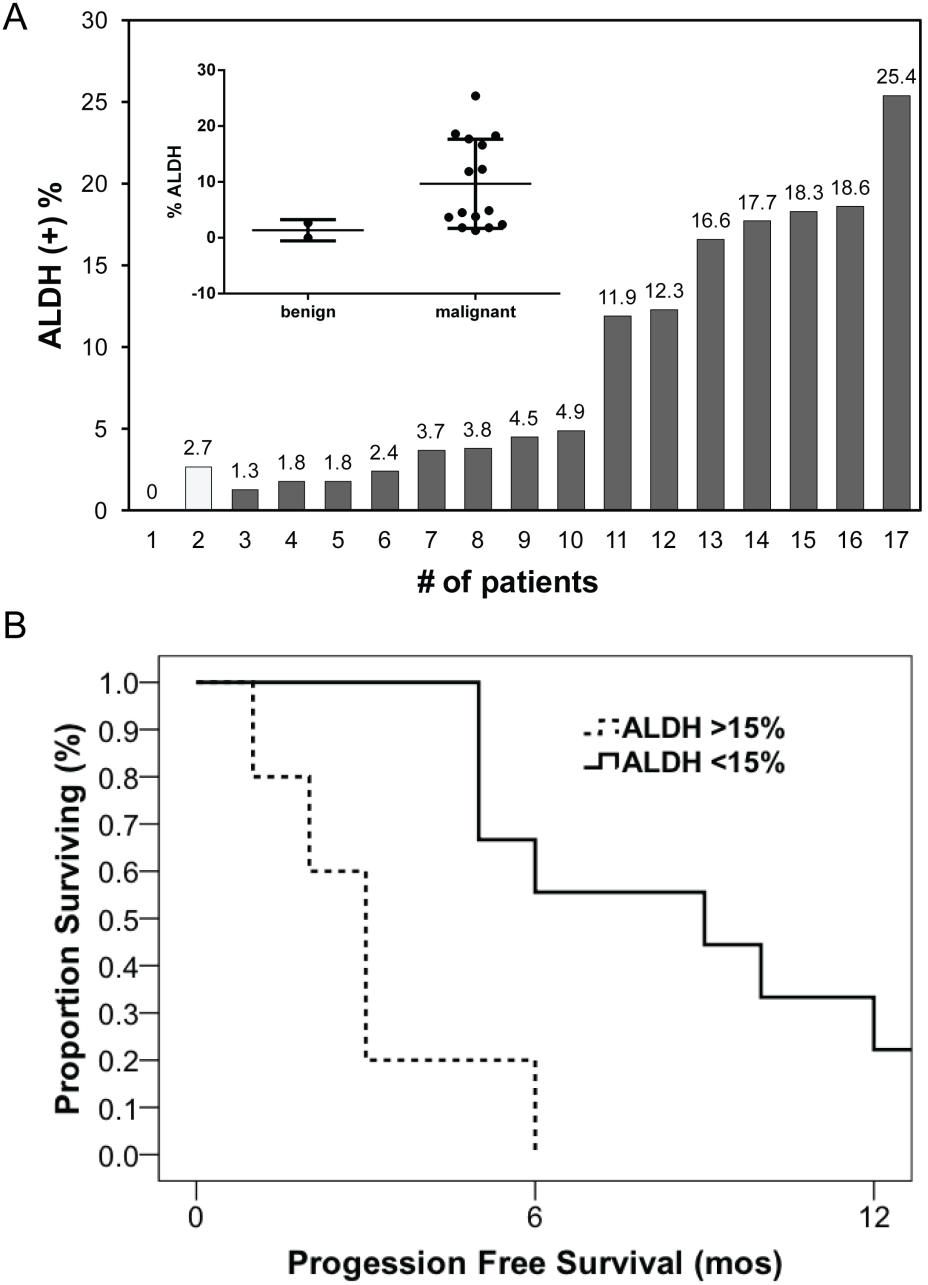
ALDH1A1 status correlates with progression free survival in ovarian cancer patients. Ascites from 15 consecutive patients with primary advanced stage III/IV ovarian cancer and 2 from benign (Miegs syndrome) was obtained and analyzed for percentage of ALDH+ cells through ALDEFLUOR assay. The histogram in inset shows the percent of ALDH+ cells in benign and malignant ascites (A). The clinicopathologic information from the ovarian cancer patients were correlated with the percent of ALDH+ cells in their ascites and evaluated progression free survival with ALDH^HIGH^ (>15% ALDH) to ALDH^LOW^ (<15% ALDH) patients (3 vs. 9 months, respectively; *p*<0.01) (B).

To assess whether ALDH+ cancer stem-like cells are also present in primary tumors and its association with progression free survival of the patients, we have collected ascites from 15 ovarian cancer patients with advanced disease and 2 benign ascites from Meigs syndrome patients and analyzed for the percent of cells with ALDH expression using ALDEFLOUR assay. In agreement with cancer stem-cell hypothesis, ALDH+ cells were present in much greater percentage in malignant ascites compared to their benign counterparts. The percentage of ALDH+ cells in malignant ascites ranged from 1.3 to 25.4% (patients 3–17) compared to 2 benign ascites (patients 1 & 2) (Figure 2A). Most importantly, the percent of ALDH+ cells in patient ascites inversely correlated to progression free survival. Patients that exhibited ALDH^HIGH^ (>15% ALDH) in their ascites demonstrated significantly lower progression free survival compared to patients with ALDH^LOW^ (<15% ALDH) (3 vs. 9 months, respectively; *p* = 0.003) (Figure 2B). Though it is difficult to draw a meaningful conclusion due to the limited number of ascites used in this study, these data indicates a direct clinical correlation between ALDH status with tumor response to platinum agents and progression free survival of the ovarian cancer patients.

### ALDH+ ovarian cancer cells exhibits stem cell-like properties

**Figure 3:**
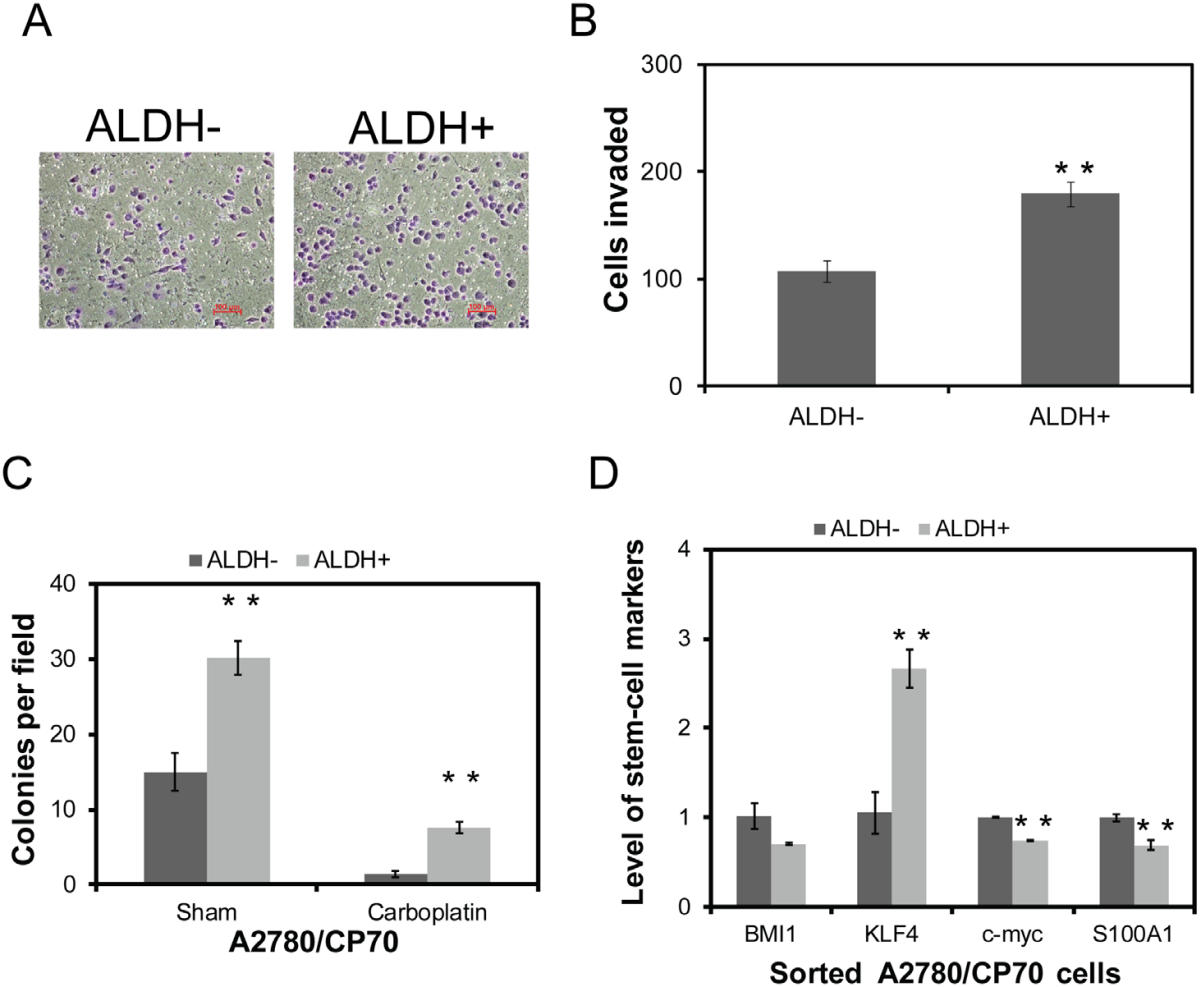
ALDH+ phenotypes demonstrate cancer stem cell properties. A2780/CP70 cells were sorted into ALDH− and ALDH+ phenotypes and evaluated for their abilities for invasion using Matrigel invasion chambers (A), quantitative data as represented (B) and colony formation potential in the presence and absence of carboplatin (20 µM) were assessed using soft agar colony formation assays (C). In order to determine possible mechanism for cancer stem cell characteristics in ALDH+ cells, we evaluated multiple markers of “stemness”. A2780/CP70 cells were sorted into ALDH− and ALDH+ phenotypes, total RNA was isolated, cDNA was prepared and real-time quantitative RT-PCR was performed (D). **indicates statistical significance (*p*<0.01).

These results led us to further characterize ALDH as a potential stem-cell marker in ovarian cancer. Tumor progression can be associated with the presence of a subset of cells that express stem cell markers and exhibit aggressive behavioral properties including invasive and increased colony formation potential. To investigate their invasive properties, sorted cells (ALDH+ vs. ALDH−) were assessed using Matrigel invasion assay. As shown in the figures 3A & 3B, ALDH+ cells demonstrated over 1.7 fold increase (*p*<0.01) in invasion through Matrigel compared to ALDH− cells.

Another surrogate measure to characterize cancer stem-like cells is the ability to form colonies, especially in the presence of chemotherapeutics [26]. Consistent with their invasive potential, ALDH+ cells demonstrated enhanced colony formation ability compared with ALDH− phenotypes (2-fold increase, *p*<0.01). Importantly, ALDH+ phenotypes displayed enhanced colony formation ability in the presence of carboplatin (5.4-fold increase, *p*<0.01) (Figure 3C). In order to gain insight into the potential stem-like mechanisms of these cells, we evaluated potential stem cell pathways of interest in these cells. The RT-PCR data showed nearly 3-fold higher level expression of Krüppel-Like Factor 4 (KLF4) in ALDH+ cells compared to their ALDH− counterparts. KLF4 belongs to zinc finger family of transcription factors, which has been reported to be critical for the maintenance of breast cancer stem cells and their aggressive behavior such as migration and invasion ad resistance to cisplatin [27] [28]. However, other stem cell mediators like BMI1, c-myc, Oct3/4 and S100A1 did not show any noticeable changes in their expression compared with ALDH− phenotypes (*p*<0.01) (Figure 3D).

### ALDH1A1 isotype promotes ovarian cancer stem-like cells’ properties

**Figure 4:**
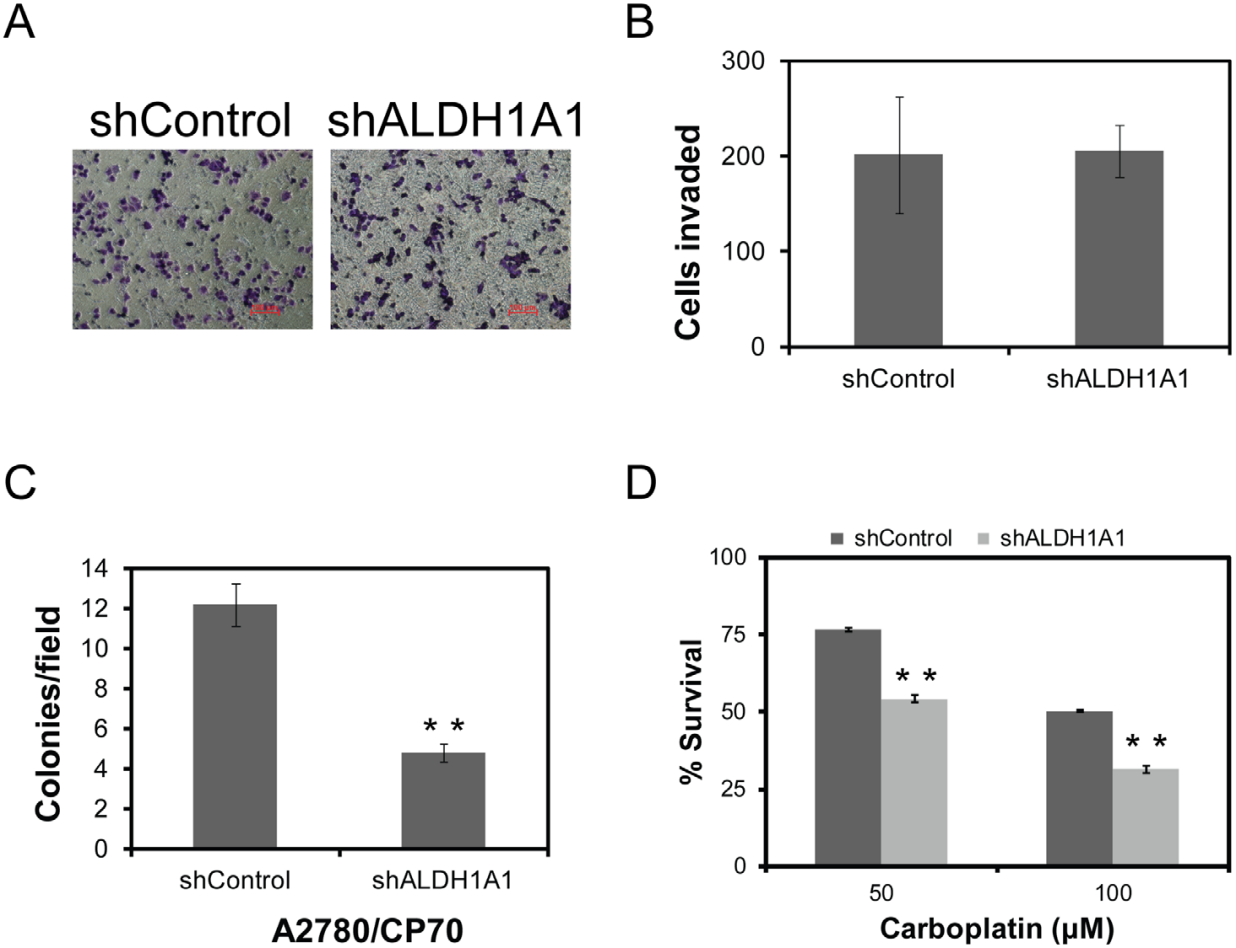
ALDH1A1 down regulation differentially affects on cancer stem cell properties. Lentiviral vectors expressing ALDH1A1 specific shRNAs or nontargeting shRNAs were transfected into A2780/CP70 cells and its effect on stem-like cell properties were evaluated for invasive potential using Matrigel invasion chambers (A), quantitative data as represented (B), clonogenic potential using soft agar colony formation (C, and carboplatin dependent growth inhibition by CellTiter-Glo Luminescent Cell Viability Assay (D). Statistical significance was evaluated using student’s *t-test.*

Consistent with the CSC literature, our data revealed elevated expression of ALDH1A1 isozyme in the platinum resistant A2780/CP70 cells. To further evaluate the role of ALDH1A1 in maintenance of cancer stem-like cells properties of platinum resistant ovarian cancer cells, we have downregulated ALDH1A1 specific isozyme using shRNAs (Figure S1). Contrary to its high expression, downregulation of ALDH1A1 has not shown any considerable differences in invasive properties of these cells (Figure 4A & 4B). However, ALDH1A1 knockdown affected their ability to form colonies in soft agar, demonstrating a 2.5-fold decrease in the colonies (*p*<0.01) (Figure 4C). Similarly, downregulation of ALDH1A1 alone significantly sensitized inherently platinum resistant A2780/CP70 cells to carboplatin. Considering IC_50_ doses required (82.9 µM and 43.8 µM for negative control and shALDH1A1 cells respectively) for these cells, about 50% reduction in carboplatin dose is evident for ALDH1A1 knockdown cells (*p*<0.001) (Figure 4D). Together, these data suggests that ALDH1A1 status is one of the critical factors in maintaining stem-like cell properties and platinum resistance in ovarian cancer.

### ALDH1A1 controls cell cycle checkpoints by regulation of KLF4 and p21 proteins

**Figure 5:**
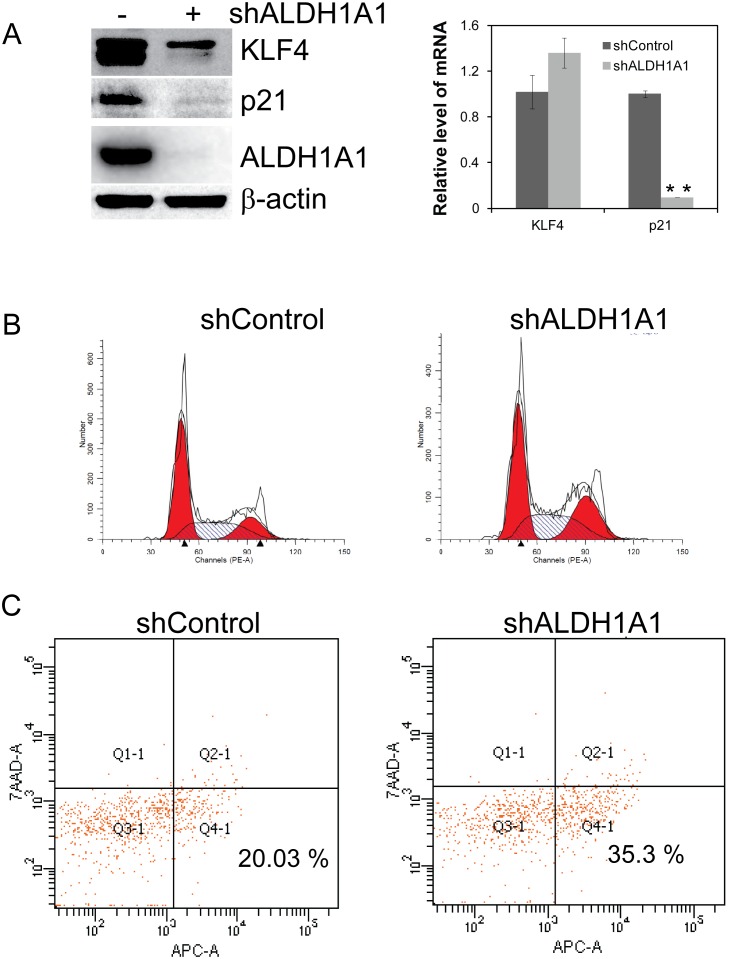
ALDH1A1 downregulation leads to lower KLF4 & p21 expression with altered cell-cycle profile. ALDH1A1 proficient and deficient A2780/CP70 cells were evaluated for expression of stem-like cell marker KLF4 and cell cycle checkpoint protein p21 by RT-PCR and Western blot analysis (A). Cell cycle distributions of cells were analyzed after fixing the cells in 70% methanol and propidium iodide staining followed by flow cytometry (B). Apoptotic cells were detected after staining the cells with APC-Annexin V and 7-AAD according to the manufacturer’s instructions and analyzed by flow cytometry (C).

Increased expression of KLF4 in ALDH+ cells (Figure 3D) led us to evaluate any functional relationship between ALDH1A1 and KLF4. Although ALDH1A1 knockdown did not affect the level of KLF4 mRNA, a significant decrease in KLF4 protein levels was evidenced in these cells (Figure 5A). Considering that the functional status of the cell cycle regulator p21 is intimately related to KLF4’s function, the mRNA and protein levels of p21 were also assessed. As expected, both ALDH1A1 transcript and protein levels of p21 were dramatically decreased in ALDH1A1 deficient A2780/CP70 cells (Figure 5A). Since ALDH1A1 status influenced expression of cell cycle checkpoint proteins p21/CDK4, we further analyzed the cell cycle profiles of these cells. The flow cytometric data clearly indicate decreased G1 cell population (50.25% to 42.10%) and a compensatory increase in accumulation of cells in S and G2 phases (Figure 5B). Due to the fact that cells in active replication (S and G2 phases) are more vulnerable to genotoxic stress compared to their non-dividing or other restive phases (G0 and G1), the re-sensitization of ALDH1A1 deficient cells may be in part attributable to the changes in cell cycle distribution. Furthermore, we have also confirmed that KLF4 status affects p21 expression levels in A2780/CP70 cells (Figure S2A & S2B). However, evaluation of ALDH activity and ALDH1A1 expression in cells did not exhibit any noticeable changes, suggesting KLF4 likely serves downstream to ALDH1A1 (Figure S2C).

To further evaluate the genes responsible for chemoresistance and cell cycle regulation based on the status of ALDH1A1, we have used RT^2^ Profiler PCR Array for the human Cancer Drug Resistance & Cell Cycle (Ambion). The comparative expression profiles of genes in ALDH1A1 knockdown vs. control cells revealed a significant decrease in expression of the cell cycle regulators CDKN1A (p21) (0.27-fold) and CDK4 (0.26-fold), which confirms its role in conjunction with KLF4 (Table 1).

**Table 1:** The human Cancer Drug Resistance & Cell Cycle RT^2^ Profiler PCR Array results were compared between ALDH1A1 knockdown and controls.

Gene	Function	Fold difference (shRNA/control)
CDKN1A (p21)	A potent cyclin-dependent kinase inhibitor(CKI); A regulator of cell cycleprogression at G1.	0.27
CDK4	A part of the cyclin-dependent kinase family;Important for cell cycle G1 phaseprogression.	0.26
BAX	Accelerating apoptosis.	3.95

Of note, with restored platinum sensitivity from ALDH1A1 knockdown, expression of the pro-apoptotic factor BAX was up-regulated nearly 3.95-fold. ALDH1A1 knockdown demonstrated increased number of cells in early apoptotic phase compared to the control. After exposure of cells to 1.0 µM staurosporine for 6 hours, a dramatic increase in early apoptotic cells was observed in ALDH1A1 deficient cells compared to their ALDH1A1 proficient counter parts (35.3% vs. 20.3%) (Figure 5C). These data suggests that ALDH1A1 knockdown cells are more susceptible to BAX-induced apoptosis, which has been well described in the inhibition of p21 cell cycle checkpoint [29].

### ALDH1A1-mediated platinum resistance correlates to altered DNA repair networks

**Figure 6:**
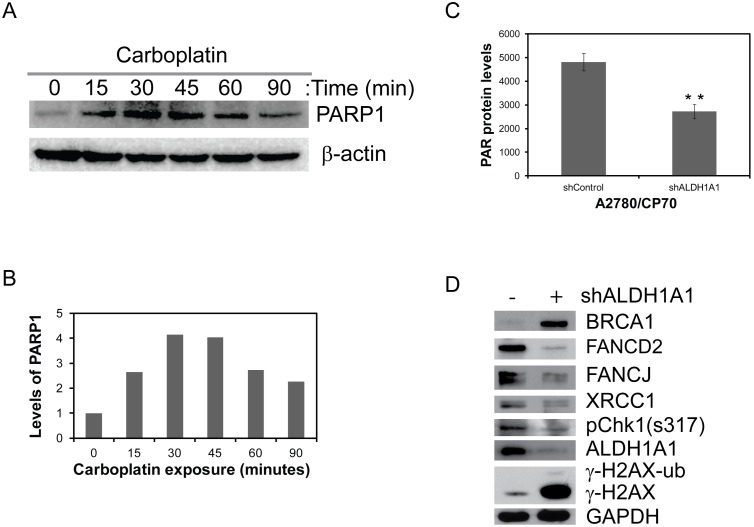
ALDH1A1 status alters cell cycle checkpoints and DNA repair networks. ALDH1A1 proficient (control) and deficient (shALDH1A1) A2780/CP70 cells were assessed for their abilities to repair carboplatin induced single strand breaks by looking at time dependent induction of PARP-1 protein levels by western blots (A), densitometry of blots by Image J (B) and total PARP activity was assayed by measuring PAR levels (C). To assess the ALDH1A1 dependent expression DDR and repair proteins, whole cell lysates were normalized for total proteins and western blot analysis were performed using antibodies as represented (D).

Intact cell cycle checkpoints and DNA damage response (DDR) signaling mechanisms are important for the cell’s ability to counter with different kinds of genomic insults and orderly progression of cell cycle. However, a common feature of cancer cells is altered regulation of these signaling cascades to acquire additional genetic changes required for re-differentiation and survival thereby display therapeutic resistance. Likewise, ALDH1A1 cells displayed altered regulation of KFL4/p21 mediated cell cycle checkpoint mechanism, which primarily directs the inhibition of G1 to S and G2 to M progression in response to DNA damage to allow more time for the cell to repair. Considering the association of PARP-1 in repair of carboplatin induced DNA damage, we evaluated its involvement in ALDH1A1 cells. PARP-1 levels progressively increased up to 45 minutes following carboplatin treatment (Figure 6A and 6B). However, downregulation of ALDH1A1 resulted in significant decrease (1.8 fold) in total PAR levels (PARP activity) (Figure 6C) compared to ALDH1A1 proficient cells (*p* = 0.012).

Several recent studies on breast cancer stem-cells indicate altered regulation of DNA repair networks, particularly an inverse relationship between ALDH1A1 status and BRCA1 gene expression [15], [16]. Moreover, in response to DNA damage, BRCA1 is known to govern cell cycle checkpoints and choice of the DNA repair pathways to timely repair of these DNA lesions [16]. Consistent with breast cancer stem-cell data, Western blot analysis revealed an inverse relationship between ALDH1A1 and BRCA1 expression in A2780/CP70 cells, suggesting ALDH1A1 expressing ovarian cancer stem-like cells more likely to lose or express low levels of BRCA1. Further analysis revealed down regulation of ALDH1A1 induced spontaneous DNA damage response by expressing γ-H2AX protein (a marker of double strand breaks). This is also coincided with the diminished levels of excision repair protein (XRCC1), replication checkpoint kinase protein 1 (Chk1) and other replication stress associated Fanconi anemia (FA)-BRCA gene products FANCD2 and FANCJ [30]–[32]. Together these data suggest that ovarian cancer stem-like cells may maintain therapeutic resistance by expressing ALDH1A1 and depletion of which, abrogates G1 and S-phase checkpoints (Figures 5A and 6C) leading to replication stress (Figure 5B). Though ALDH1A1 depleted cells accumulated in S and G2 phases, the phosphorylation status of replication checkpoint protein Chk1 (Ser317) and expression of replication fork associated FA pathway proteins FANCD2 and FANCJ were affected. This is also conferred by induction of γ-H2AX, a marker for DSB and reduced cell survival. Since Chk1 and FA proteins are important for the replication checkpoint and the stability of stalled replication forks, the spontaneous DNA damage response in these cells can be attributed to defect in these proteins (Figure 6D). To identify the molecular network signals that are differentially expressed in ALDH1A1 expressing cells, we initially assessed the expression of Fanconi anemia pathway and tumor resistance to chemotherapeutic agents. Consistently, our results demonstrated a direct correlation between ALDH1A1 status and FANCD2 expression. Together our data indicates a connection between ALDH1A1 status to stemness and platinum resistance of ovarian cancer cells by altered regulation of DNA repair works.

## Discussion

Several potential ovarian CSCs specific surface markers have been described such as CD44+/CD117+ [33], CD44+/MyD88+ [34], CD133+ [35], CD44+/CD24− [21], [36] and ALDH/CD133+ [37]. Detecting ALDH1A1 via the ALDEFLUOR assay is a simple and effective approach for identifying and isolating ovarian CSCs from cell lines and primary tissues. Importantly, its detection via a functional assay of stem-cells is advantageous opposed to surface markers that might or might not be actively contributing to stem cell features. Considering the assay of ALDH+ cells are based on their fluorescence, they remain viable and amenable to further in vitro and in vivo research as well as clinical applications.

We demonstrated that ALDH+ phenotypes exhibit cancer stem-like properties of enhanced invasion, colony formation ability, as well as increased expression of stem cell mediator KLF4. Additionally, our data confirmed that platinum resistant cell line A2780/CP70 exhibits much higher ALDH activity than its isogenic parental platinum sensitive cell line A2780. Importantly, presence of ALDH+ cells also associates with clinical and pathological relevance of tumor ascites, with a direct correlation to worse progression free survival.

ALDH and its expression have been linked to poor prognosis in several cancer models [4]. Particularly, ALDH1A1 isozyme has been shown to play an important functional role in maintaining cancer stem cells. In this study, we further investigated the potential role of ALDH1A1 isozyme in maintenance of ovarian cancer stem-like cells’ properties. The stable downregulation of ALDH1A1 isozyme alone dramatically decreased their ability to form colonies. Although ALDH+ cells demonstrated increased invasive properties compared to ALDH− cells, a difference in invasive potential of a single isozyme ALDH1A1 was not seen. This may be attributed to invasive roles for other isoforms of ALDH and other cancer stem cell markers in maintaining certain properties of stem-like cells [38]–[41].

In regards to platinum-resistance, ALDH1A1 silencing alone sensitized the inherently platinum resistant A2780/CP70 cells to carboplatin. Further exploration of possible chemoresistance pathways in ALDH1A1 positive cells revealed a vital role for KLF4/p21 interaction. KLF are transcriptional regulators that influence several cellular functions, ranging from differentiation to proliferation and apoptosis. Being a potent inhibitor of cell cycle progression, p21 seems to be intimately related to KLF4’s function; KLF4 and p21 are context-dependent opposing force in cancer [42]. We found that ALDH1A1 silencing leads to diminished levels of both KLF4 and p21. Yu et al [27] reported knockdown of KLF4 in breast cancer cells decreased the proportion of stem/progenitor cells as demonstrated by expression of stem cell surface markers ALDH. In this study, KLF4 knockdown in A2780/CP70 cells could lead to decreased expression of p21, but did not affect ALDH activity or ALDH1A1 expression, suggesting differential regulation of stem-cell pathways.

This KLF4/p21 mediation has been well described in the literature with numerous publications demonstrating its ability to control chemoresistance [43], [44]. ALDH1A1 knockdown cells show reduced expression of p21 and cyclin-dependent kinase 4 (CDK4). CDK4 is one of the members of cyclin-dependent kinase family while p21 is a potent CDK inhibitor. Both p21 and CDK4 regulate cell cycle progression at G1 phase progression [45] and ALDH1A1 silencing induces A2780/CP70’ cell-cycle arrest (S and G2 arrest) which is a more favorable phase for genotoxins induced cell death. Importantly, p21 and CDK4 levels were decreased with knockdown of ALDH1A1, and this was also associated with BAX-mediated apoptosis, where a 4-fold increase in BAX levels was observed (Table 1).

Cancer stem cells may maintain their stem-ness by altered expression of cell cycle checkpoints such that they can become resistant to therapies due to their accumulation at particular cell cycle phase. In response to DNA damage, altered cell cycle and checkpoint signals also differentially regulate DNA repair networks. In our study, consistent with G1-phase accumulation, ALDH1A1 proficient cells exhibited increased expression of G1 checkpoint proteins KLF4/p21 and CDK4. Concomitant downregulation of ALDH1A1 exhibited increased accumulation of cells in S-phase but decreased S-phase checkpoint leading to DDR as evidenced by γ-H2AX. Likewise, when KLF4 was transiently downregulated in these cells, decreased levels of p21 and CDK4 was observed (Figure S2B). Further systematic evaluation of ALDH1A1/cell cycle axis is needed to confirm the platinum resistance and poor prognosis of ALDH1A1 positive ovarian cancers. Consistent with the altered cell cycle profiles and checkpoint proteins in ALDH1A1 cells, our studies also demonstrated differential expression of DNA repair network proteins. Although depletion of ALDH1A1 led to G1 checkpoint abrogation and increased S-phase accumulation of the cells, the replication checkpoint (pChk1) and replication stress associated DDR proteins FANCD2 and FANCJ were drastically diminished. Conversely, ALDH1A1 depletion in A2780/CP70 cells resulted in robust increase of BRACA1 protein in association with γ-H2AX induction, demonstrating altered regulation of DNA damage response and repair networks in cancer stem-like cells.

Collectively, these results indicate ALDH over-expression is associated with many properties of ovarian cancer stem-like cells such as enhanced invasion, colony formation, and chemoresistance. Our studies also demonstrated that ALDH1A1 plays a key role in maintenance of ovarian cancer stem-like cells’ properties and might mediate carboplatin resistance through altered regulation of cell cycle and DNA repair networks. These new findings offer an important tool for the study of ovarian CSCs and provide a potential prognostic factor and therapeutic target for treatment of patients with ovarian cancer. Despite the fact these results do not explain the mechanistic basis for these altered cell cycle and DNA repair networks, this pilot study reveal an important features of chemoresistance mechanisms adopted by ovarian cancer stem-like cells. Importantly, our data also implicates a novel connection between ALDH1A1 status and altered regulation of cell cycle and DNA repair networks that influences on ovarian cancer stem-like cell properties and platinum resistance. However, molecular basis by which ALDH1A1 regulates cell cycle checkpoint signaling and DNA repair networks needs to be evaluated. Due to their therapeutic importance, further evaluation of molecular networks that govern these DNA repair networks based on ALDH1A1 status is urgently needed.

## Supporting Information

Figure S1 Optimization of shRNA against ALDH1A1 in A2780/CP70 cells. Six different pGIPZ Lentiviral shRNA vectors against ALDH1A1 as well as negative control shRNA were transfected into A2780/CP70 cells, respectively. 0.8 µg/ml puromycin was used to select the transfected cells to decrease the background (A). After optimization through real-time quantitative RT-PCR and Western Blot, the ALDH1A1 vector 398453 demonstrated efficient transfection as well as superior knockdown efficacy (B and C).
(TIF)

Figure S2 KLF4 silencing led to significantly decreased p21, without affecting ALDH. A2780/CP70 cells were plated in 6-well plates for 18–24 hours before transfection. 60 pmols of siRNA against KLF4 as well as scramble siRNA was transfected into A2780/CP70 cells through Lipofectamine 2000 reagent (Invitrogen). 36 hours later, cells were harvested to detect KLF4, p21, ALDH1A1 expression through Western Blot and ALDH activity using ALDEFLUOR assay. KLF4 knockdown through siRNA led to significantly lower level of p21 (A and B), but didn’t affect ALDH activity or ALDH1A1 expression in A2780/CP70 cells (B and C).
(TIF)
