# Supplementary figures and images for: ALDH1A1 Maintains Ovarian Cancer Stem Cell-Like Properties by Altered Regulation of Cell Cycle Checkpoint and DNA Repair Network Signaling
Source: PLoS One. 2014 Sep 12;9(9):e107142. doi: 10.1371/journal.pone.0107142 (PMC4162571; doi:10.1371/journal.pone.0107142)

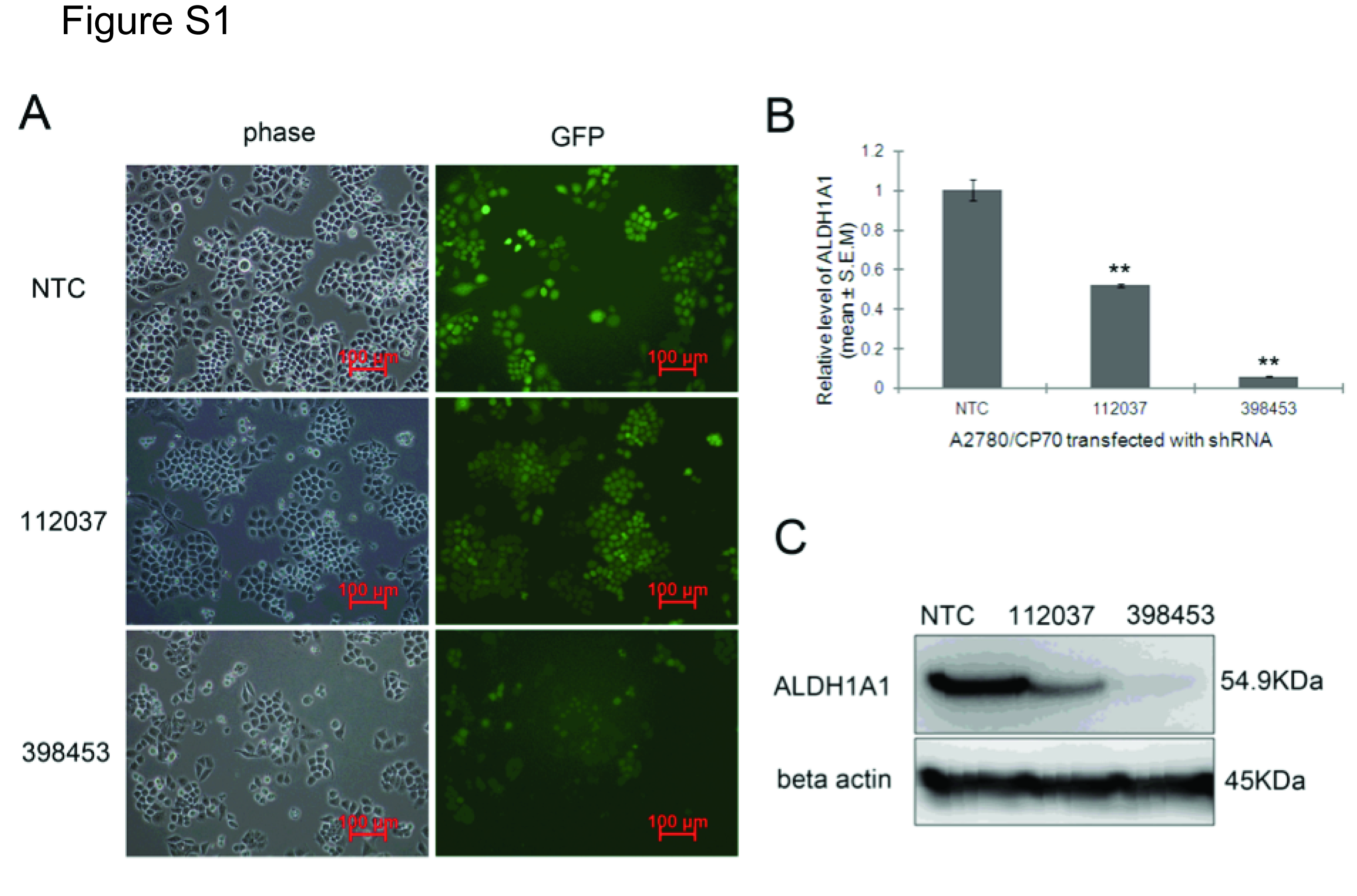

Supplement: Figure S1 — Optimization of shRNA against ALDH1A1 in A2780/CP70 cells. Six different pGIPZ Lentiviral shRNA vectors against ALDH1A1 as well as negative control shRNA were transfected into A2780/CP70 cells, respectively. 0.8 µg/ml puromycin was used to select the transfected cells to decrease the background (A). After optimization through real-time quantitative RT-PCR and Western Blot, the ALDH1A1 vector 398453 demonstrated efficient transfection as well as superior knockdown efficacy (B and C). (TIF) [file pone.0107142.s001.tif]

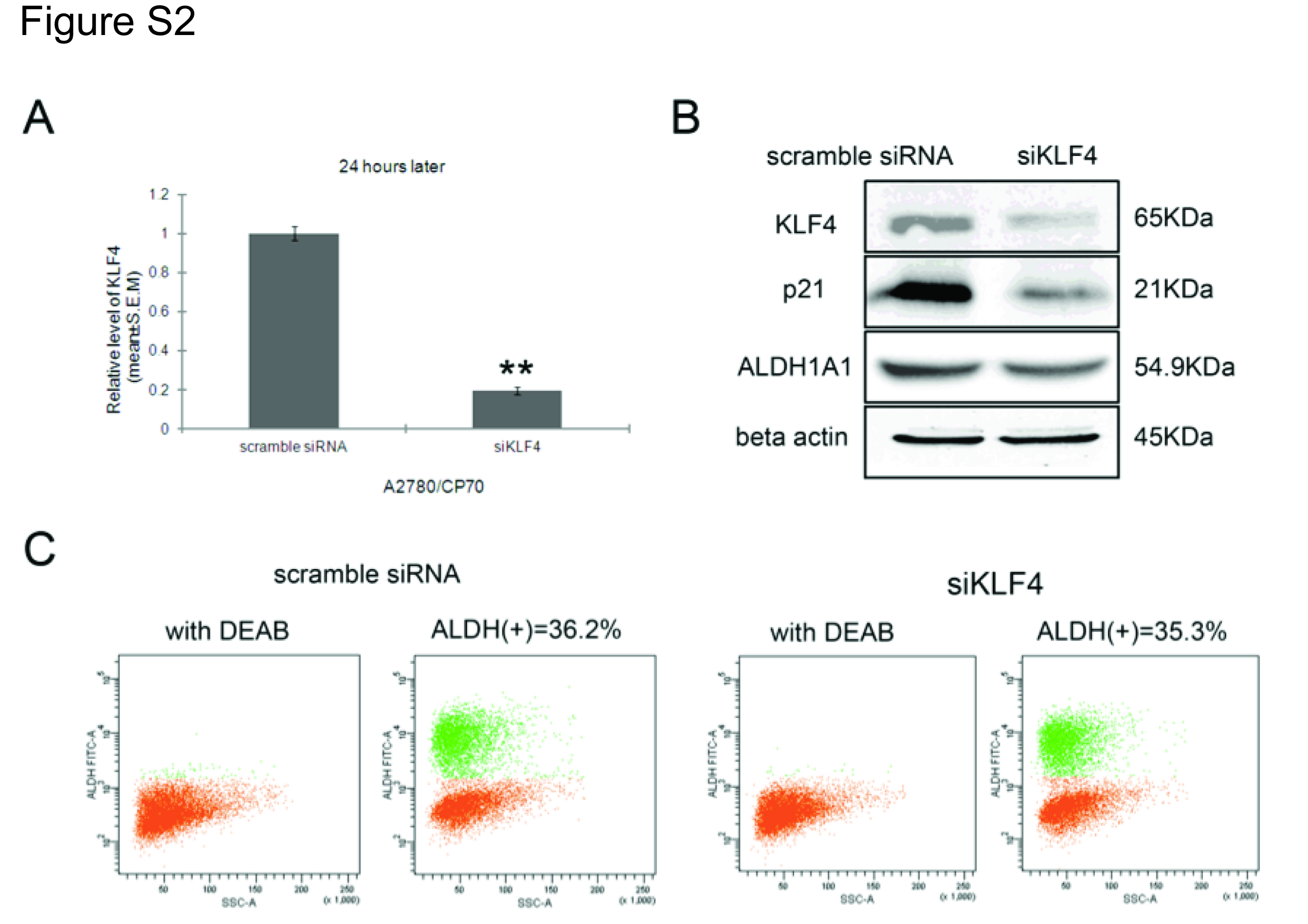

Supplement: Figure S2 — KLF4 silencing led to significantly decreased p21, without affecting ALDH. A2780/CP70 cells were plated in 6-well plates for 18–24 hours before transfection. 60 pmols of siRNA against KLF4 as well as scramble siRNA was transfected into A2780/CP70 cells through Lipofectamine 2000 reagent (Invitrogen). 36 hours later, cells were harvested to detect KLF4, p21, ALDH1A1 expression through Western Blot and ALDH activity using ALDEFLUOR assay. KLF4 knockdown through siRNA led to significantly lower level of p21 (A and B), but didn’t affect ALDH activity or ALDH1A1 expression in A2780/CP70 cells (B and C). (TIF) [file pone.0107142.s002.tif]
